# A Neuropathy Mimic: Statin-Induced Myopathy Presenting as Guillain-Barré Syndrome

**DOI:** 10.7759/cureus.66483

**Published:** 2024-08-08

**Authors:** Varsha Shinde, Pranay Penmetsa, Karthik R Nair, Yash Dixit

**Affiliations:** 1 Emergency Medicine, Dr. D. Y. Patil Medical College, Hospital and Research Centre, Dr. D. Y. Patil Vidyapeeth (Deemed to be University), Pune, IND

**Keywords:** guillain-barré syndrome (gbs), statin-induced myositis, rhabdomyolysis, statin-induced myopathy, statins

## Abstract

Statins are widely used to manage dyslipidemia and prevent cardiovascular diseases due to their effectiveness and general safety profile. However, they can sometimes cause severe muscle-related adverse effects, presenting diagnostic challenges when symptoms overlap with other conditions. This case report describes a middle-aged woman who presented to the emergency department with bilateral lower limb weakness, initially suggesting Guillain-Barré syndrome (GBS). Despite her history of low-grade fever and diarrhea, primary and secondary surveys, including electrocardiogram, blood gas analysis, and nerve conduction studies, showed no definitive signs of GBS. The patient had a recent history of percutaneous transluminal coronary angioplasty and was on dual antiplatelet therapy and rosuvastatin. Elevated creatine kinase levels and exclusion of other differential diagnoses led to the diagnosis of statin-induced myopathy, a rare but severe adverse effect of statins. The patient was treated with intravenous fluids, cessation of statins, and sessions of hemodialysis and plasmapheresis, resulting in significant improvement and eventual recovery of muscle power and neurological function. This case highlights the importance of recognizing statin-induced myopathy in patients with muscle weakness and emphasizes the need for thorough clinical evaluation to differentiate it from other conditions such as GBS. Further research is warranted to understand the pathophysiology of statin myopathy and identify at-risk populations.

## Introduction

Statins are drugs used to treat dyslipidemia. They inhibit the enzyme 3-hydroxy-3-methyl-glutaryl-coenzyme A (HMG-CoA) reductase, helping to lower serum cholesterol, triglycerides, low-density lipoprotein, and very-low-density lipoprotein levels. Statins are well-tolerated and effective in preventing cardiovascular diseases and reducing mortality and long-term morbidity in patients with coronary artery disease [1]. Despite their benefits, statins can have adverse effects, with the most common being constipation, headache, nausea, and vomiting. Some patients may experience muscle-related adverse effects. A meta-analysis of randomized controlled trials found that statins increased the risk of myopathy by 0.05% per year compared to placebo. This translates to about five additional cases per 10,000 person-years of statin therapy [2].

Risk factors for statin-induced myopathy include female sex, advanced age, vigorous exercise, and multisystemic diseases such as diabetes and hypothyroidism [3]. The incidence of statin-induced myopathy varies among different statins, with more lipophilic statins such as atorvastatin more likely to produce muscle symptoms than hydrophilic statins such as rosuvastatin [4]. Muscle-related adverse events range from benign myalgia to life-threatening necrotizing myositis with rhabdomyolysis and renal failure [5]. Muscle symptoms are more common in proximal muscles and worsen with movement or exercise [5].

Guillain-Barré syndrome (GBS) is a rare but serious immune-mediated neuropathy associated with acute-onset, flaccid neuromuscular paralysis [6]. Animal studies have shown molecular mimicry between the bacterial lipoproteins of *Campylobacter jejuni* and host nerves, with antibodies against the bacterial lipoprotein attacking the host nerves [7]. Patients usually present with symmetrical, ascending flaccid muscle weakness following an antecedent illness four to six weeks earlier. This case report highlights a rare presentation of statin-induced myopathy with signs and symptoms mimicking GBS and discusses the treatment modalities used.

## Case presentation

A middle-aged woman presented to the emergency department with a history of bilateral lower limb weakness for two days. On a primary survey, her airway was patent, respiratory rate was 16 breaths per minute with 97% saturation on pulse oximetry, heart rate was 88 beats per minute, and blood pressure was 118/70 mmHg in the right arm in a supine position. She was fully conscious and oriented with a Glasgow Coma Scale score of 15/15, and her capillary blood glucose was within reference limits.

Primary adjuncts were performed, including chest X-ray, electrocardiogram (ECG), and blood gas analysis. The ECG indicated tall T waves, suggesting hyperkalemia, while blood gas analysis revealed metabolic acidosis with hyperkalemia. The chest X-ray was unremarkable. The patient was immediately treated with anti-hyperkalemic measures, including calcium gluconate, insulin drip, and salbutamol nebulization.

On a secondary survey, the patient reported a history of low-grade fever for 10 days, accompanied by multiple episodes of watery diarrhea. After consulting a clinic and receiving treatment, her fever and diarrhea subsided. A few days later, she developed bilateral painless muscle weakness in her lower limbs, starting from her calf muscles and progressing to her thighs. She became immobile, lost bowel and bladder control, and had significant muscle weakness in her lower limbs (power: 1/5). Her deep tendon reflexes were absent, and plantar reflexes were mute bilaterally. Muscle tone was normal. Examination of the upper limbs revealed weakness, more pronounced in the left upper limb (power: 4/5 in the right upper limb and 3/5 in the left upper limb). Sensation was absent in the bilateral lower limbs up to the thighs. The single breath count was 15 seconds, and the neck holding time was 35 seconds. She had a history of percutaneous transluminal coronary angioplasty to the left anterior descending artery four months ago and had been on dual antiplatelet drugs and rosuvastatin (20 mg) since then.

Differential diagnoses included GBS, transverse myelitis, statin-induced myopathy with rhabdomyolysis, and epidural abscess. Blood cultures showed no growth, stool routine and microscopy were normal, and stool culture showed no growth. Relevant blood investigations are presented in Table 1.

**Table 1:** Patient laboratory values. AST: aspartate aminotransferase; ALT: alanine aminotransferase; CRP: C-reactive protein; CSF: cerebrospinal fluid; ESR: erythrocyte sedimentation rate; INR: international normalized ratio; NA: not applicable; SGOT: serum glutamic-oxaloacetic transaminase; SGPT: serum glutamic-pyruvic transaminase; WRL: within reference limits

Analyte	Patient value	Reference range
Hemoglobin	12.5 g/dL	13.2–16.6 g/dL
Total leukocyte count	12,700/µL	4,000–10,000/µL
Platelet count	352,000/µL	150,000–410,000/µL
Total bilirubin	0.57 mg/dL	0.22–1.2 mg/dL
Conjugated bilirubin	0.25 mg/dL	<0.5 mg/dL
Unconjugated bilirubin	0.32 mg/dL	0.1–1.0 mg/dL
SGOT (AST)	325 U/L	8–48 U/L
SGPT (ALT)	363 U/L	7–55 U/L
Alkaline phosphatase	84 U/L	40–129 U/L
Urea	273 mg/dL	17–49 mg/dL
Creatinine	11.24 mg/dL	0.6–1.35 mg/dL
Sodium	131 mmol/L	136–145 mmol/L
Potassium	6.7 mmol/L	3.5–5.1 mmol/L
Calcium	7.8 mg/dL	8.6–10.2 mg/dL
Magnesium	3.2 mg/dL	1.8–2.4 mg/dL
Phosphorus	>9 mg/dL	3.5–4.5 mg/dL
Uric acid	14.2 mg/dL	NA
Prothrombin time	13.5 seconds	10.09–13.79 seconds
INR	1.13	0.85–1.15
ESR	87 mm/hour	<20 mm/hour
CRP	27.7 mg/L	<5 mg/dL
CSF routine microscopy	WRL	NA
Creatine phosphokinase	14,566 U/L	25–190 U/L
Protein	Trace	>40 mg/dL
Pus cells	1–2 cells/hpf	<5 cells/hpf
Urine myoglobin	Positive	NA

Magnetic resonance imaging of the brain and whole spine was unremarkable. Nerve conduction studies, performed due to suspicion of GBS, were normal. Given the patient’s history of statin use, elevated creatine kinase levels, and exclusion of other diagnoses, a final diagnosis of statin-induced myopathy was made. The patient received intravenous fluids for hydration, statins were discontinued, and dual antiplatelet therapy was continued. She was admitted to the intensive care unit (ICU), where she underwent two sessions of hemodialysis followed by two sessions of plasmapheresis due to suspected anti-HMG-CoA reductase antibodies in her plasma. Unfortunately, testing for anti-HMG-CoA reductase antibodies was not available in the city. Following treatment, the patient began to regain strength and sensation in her limbs. After 15 days in the ICU, she was transferred to the ward and subsequently started on oral steroids. She was discharged after 35 days of hospitalization with full recovery of muscle power (5/5) in all limbs and regained bowel and bladder control. She was started on atorvastatin on discharge and was advised regular follow-ups in the outpatient department.

## Discussion

Statins, or HMG-CoA reductase inhibitors, are the most successful class of drugs used to treat dyslipidemia, which plays a role in the pathogenesis of coronary artery disease and atherosclerosis. These medications have been in use for decades and are safe, well-tolerated, and frequently prescribed for atherosclerosis. However, like all drugs, statins have adverse effects, ranging from benign muscle pain and weakness to life-threatening rhabdomyolysis. If not identified early, these adverse effects can increase mortality and morbidity.

The pathophysiology of statin-induced myopathy is not well established, but several theories have been proposed. Cholesterol synthesis involves multiple steps, with the conversion of HMG-CoA into mevalonate by the HMG-CoA reductase enzyme being key. Statins block this enzyme. Skeletal membrane cholesterol modulates membrane fluidity, and altering it can affect ion channels, especially chloride channels, which control membrane depolarization. Chronic statin use can lead to membrane hyperexcitability associated with impaired chloride conductance [8]. Additionally, statins may cause a deficiency of coenzyme Q10, an important mitochondrial antioxidant. Chronic statin use increases oxidative stress on mitochondria, leading to muscle cell death and the release of toxins into the bloodstream [9].

Clinical manifestations of statin-induced myopathy include mild muscle weakness, muscle pain, myositis, and severe complications such as rhabdomyolysis. Patients usually present with proximal muscle weakness involving bilateral extremities, often with functional loss, such as difficulty raising arms above the head or getting up from a seated position. Other symptoms include muscle stiffness, tendon pain, and nocturnal cramping. On examination, there may be tenderness in the affected muscle group.

Numerous causes of muscle pain and weakness are more common than statin-induced myopathy. Distinguishing among these causes relies on the onset of muscle symptoms after introducing statins. Muscle biopsy is not highly diagnostic, as timing is critical. Early biopsies may show myocyte necrosis without vasculitis, while delayed biopsies may show mononuclear cell infiltration, indicating a repair process [10,11]. Elevated creatine kinase levels are common in muscle diseases and can occur in hypothyroidism or muscle injury from contact sports such as hockey, football, and boxing [12]. Creatine kinase levels elevated more than 10 times the upper limit, combined with myalgia symptoms and statin use, should raise suspicion of statin myopathy.

The primary management for statin-induced myopathy is the cessation of the offending drug. Statin discontinuation has been shown to reverse adverse effects without further treatment in mild cases. In cases of statin-induced rhabdomyolysis, careful monitoring is required. Patients must be well hydrated to enhance the excretion of myoglobin, which is toxic to nephrons and can lead to acute renal failure. In this case, the patient required urgent hemodialysis due to acute renal failure. After hemodialysis, the patient showed improved renal function and good urine output.

GBS was a close differential diagnosis in our patient due to symptom overlap and the absence of deep tendon reflexes. However, nerve conduction studies were unremarkable, and cerebrospinal fluid studies did not show albumin-cytological dissociation. Therefore, we concluded that statin-induced myopathy was the most probable diagnosis, confirmed by the resolution of symptoms and full neurological recovery after statin cessation.

## Conclusions

This case presented a diagnostic dilemma favoring GBS due to the history and presentation of ascending muscle weakness with areflexia. Appropriate investigations ruled out GBS, leading to a diagnosis of statin-induced myopathy after excluding other causes of acute muscle weakness. This case report highlights the overlap of symptoms between different diseases and emphasizes the importance of thorough patient history, physical examination, and relevant investigations before reaching a diagnosis. Further clinical studies are needed to better understand the pathophysiology of statin myopathy and identify those at risk.
